# Complete Chloroplast Genome of *Megacarpaea megalocarpa* and Comparative Analysis with Related Species from Brassicaceae

**DOI:** 10.3390/genes15070886

**Published:** 2024-07-05

**Authors:** Zhuo Zhang, Xiaojun Shi, Haowen Tian, Juan Qiu, Hanze Ma, Dunyan Tan

**Affiliations:** Xinjiang Key Laboratory for Ecological Adaptation and Evolution of Extreme Environment Biology, College of Life Sciences, Xinjiang Agricultural University, Urumqi 830052, China; zhangzhuo610431@163.com (Z.Z.); shixj@xjau.edu.cn (X.S.); tianhw5209@foxmail.com (H.T.); xjqiujuan@163.com (J.Q.); hanzema@163.com (H.M.)

**Keywords:** Megacarpaeeae, selection pressure, phylogeny, divergence times, SBS

## Abstract

*Megacarpaea megalocarpa*, a perennial herbaceous species belonging to the Brassicaceae family, has potential medicinal value. We isolated and characterized the chloroplast (cp) genome of *M. megalocarpa* and compared it with closely related species. The chloroplast genome displayed a typical quadripartite structure, spanning 154,877 bp, with an overall guanine–cytosine (GC) content of 36.20%. Additionally, this genome contained 129 genes, 105 simple sequence repeats (SSRs), and 48 long repeat sequences. Significantly, the *ycf1* gene exhibited a high degree of polymorphism at the small single copy (SSC) region and the inverted repeat a (IRa) boundary. Despite this polymorphism, relative synonymous codon usage (RSCU) values were found to be similar across species, and no large segment rearrangements or inversions were detected. The large single copy (LSC) and SSC regions showed higher sequence variations and nucleotide polymorphisms compared to the IR region. Thirteen distinct hotspot regions were identified as potential molecular markers. Our selection pressure analysis revealed that the protein-coding gene *rpl20* is subjected to different selection pressures in various species. Phylogenetic analysis positioned *M. megalocarpa* within the expanded lineage II of the Brassicaceae family. The estimated divergence time suggests that *M. megalocarpa* diverged approximately 4.97 million years ago. In summary, this study provides crucial baseline information for the molecular identification, phylogenetic relationships, conservation efforts, and utilization of wild resources in *Megacarpaea*.

## 1. Introduction

Brassicaceae is a large group of angiosperms, consisting of 52 tribes, 321 genera, and approximately 4000 species. They are distributed worldwide, except for Antarctica, mainly in temperate regions [1,2]. Most of the Brassicaceae plants have significant economic and medicinal value [3] and are utilized as adjuvant therapy to treat major illnesses such as cancer [4,5]. For instance, *Brassica oleracea* has been demonstrated to reduce the risk of bladder cancer, as well as other types of cancer and cardiovascular disease incidence [6]. However, taxonomic classification has remained controversial in Brassicaceae due to the utilization of various molecular markers. Previous studies categorized the phylogenetic relationships of Brassicaceae into four lineages (basal LI–III and expanded LII) [2,7] or six major clades (A–F) [8], based on the internal transcribed spacer of nuclear ribosomal DNA (ITS) or single-copy nuclear markers; however, statistical support was generally low. Subsequent studies utilizing chloroplast DNAs and nuclear genes identified five strongly supported lineages (LI–V) [9], but some tribes remained unassigned to any lineage. Therefore, phylogenetic relationships within and between these lineages in Brassicaceae still need to be further studied.

**Figure 1:**
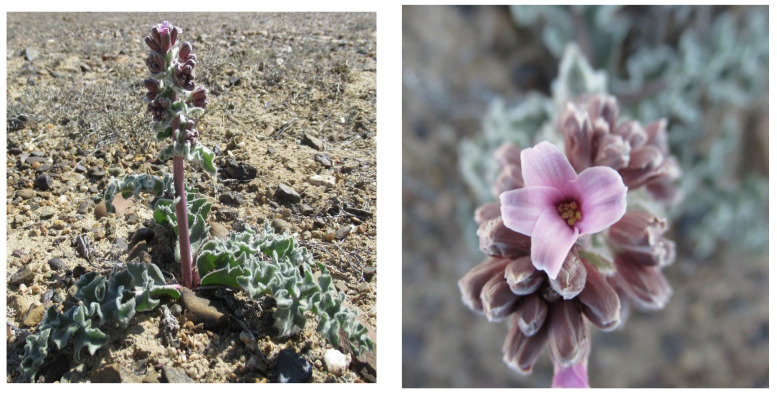
Plant characteristics of *M. megalocarpa*.

*Megacarpaea* is a perennial herb that is primarily found in Central Asia and the Himalayan region. In China, there are three species, four varieties, and one variant of *Megacarpaea* [10]. *Megacarpaea* species hold significant value as traditional medicinal plants. For example, *Megacarpaea delavayi* is renowned for its heat-clearing and stomachic effects, and the rhizomes of *Megacarpaea polyandra* are utilized as a coolant in fever treatment or as an antidote for scorpion stings and snake bites by Bai and Tibetan people [11,12]. *Megacarpaea megalocarpa* (Fisch. ex DC.) Schischk. ex B. Fedtsch., a perennial herb, grows in desert areas at altitudes of 200–3600 m. It grows 20–40 cm tall, with erect stems, and its basal leaves have oblanceolate leaf blades with pinnatisect margins. The inflorescences are paniculate, with sessile bracts at branching. The petals of *M. megalocarpa* are lavender in color (Figure 1). This species is found in the sandy deserts and alkaline plains in Kazakhstan, Kyrgyzstan, Russia, Uzbekistan, and China (Qinghai and Xinjiang). *M. megalocarpa*, a congener, may hold promise for its medicinal value. Previous studies have classified the phylogenetic relationships of *Megacarpaea* in the Brassicaceae family as part of the extended lineage II or clade C [7,13]. However, recent research has left *Megacarpaea* unassigned to any specific lineage [9], making it difficult to determine its exact phylogenetic position.

Contrary to the mitochondrial and nuclear genomes, the chloroplast genome exhibits high conservation, characterized by a slow variation rate, maternal inheritance, and sequence stability [14,15]. As a result, the chloroplast genome is extensively utilized for reconstructing phylogenetic analyses in angiosperms, identifying species, and determining the origin and divergence timelines of species [16]. Nonetheless, research on the chloroplast genomes of *Megacarpaea* species has been limited, primarily focusing on *M. polyandra* and *M. delavayi*. There have been no reports on the chloroplast genomes of *M. megalocarpa*, which impedes our understanding of phylogenetic relationships within the *Megacarpaea* genus. Hence, we propose the hypothesis that the chloroplast genome of *M. megalocarpa* exhibits similar characteristics to other *Megacarpaea* species, and the *M. megalocarpa* phylogenetic relationship is more closely related to *M. delavayi*. Consequently, this research aims to sequence, assemble, and analyze *M. megalocarpa* through high-throughput sequencing to uncover its gene-level features. Our primary goals are as follows: (1) to characterize and compare the chloroplast genome of the tribe Megacarpaeeae, including *M. megalocarpa*, *M. delavayi* (GenBank ID: KX886349), *M. polyandra* (MK637758), *Pugionium cornutum* (KT844941), *Pugionium dolabratum* (KT844940), and *Pugionium pterocarpum* (MK637779); (2) to examine simple sequence repeats (SSRs) and repeat structures in the whole cp genome of Megacarpaeeae to provide markers for phylogenetic and genetic studies; and (3) to explore the phylogenomic position of *M. megalocarpa*. This study will offer a molecular foundation for the species identification of *M. megalocarpa* and the genetic evolution of *Megacarpaeeae* species.

## 2. Materials and Methods

### 2.1. Survey Site Sampling and DNA Sequencing

Fresh, healthy leaf specimens of *M. megalocarpa* were collected from the desert region near Dure Town (88°32′15″ E,46°30′36″ N), Altay, Xinjiang. Following collection, all leaves were promptly preserved in liquid nitrogen and stored in a −80 °C ultra-low temperature freezer. The leaf samples were then dispatched to Genepioneer Biotechnologies (Nanjing, China) for genetic sequencing. DNA extraction was carried out using the Plant Genomic DNA Kit (Tian gen Biotechnology, Beijing, China). Paired-end libraries with insert sizes of 350 bp were created according to Illumina’s standard protocol for genomic DNA library preparation, with subsequent quality assessments to verify sequencing precision. The whole genome of *M. megalocarpa* was sequenced on the Illumina Novaseq 6000 PE150 platform (Illumina, San Diego, CA, USA), utilizing the sequencing by synthesis (SBS) technology.

### 2.2. Chloroplast Genome Assembly and Annotation Analyses

High-quality clean reads were obtained by utilizing Trimmomatic v.0.39 [17] to filter out sequences with low quality (where the quality value was Q ≤ 5 and N bases > 5%). The assembly of the *M. megalocarpa* chloroplast genome was conducted using the GetOrganelle v.1.7.5 [18] pipeline, with the *M. delavayi* chloroplast genome serving as the reference. Automatic annotations of the chloroplast genomes were performed by CpGAVAS2 [19] and subsequently manually curated with reference to previously published chloroplast genomes using Geneious v.2021.1.1 [20]. The structure maps of the chloroplast genome were visualized using the OGDRAW v.1.3.1 [21] online tool (https://chlorobox.mpimp-golm.mpg.de/OGDraw.html, accessed on 20 December 2023). The chloroplast genome sequence for *M. megalocarpa* was submitted to the NCBI platform under accession number PP234616.

### 2.3. Analysis of SSRs and Repetitive Sequences

The online program MIcroSAtellite (MISA) v.2.1 [22] was used to detect microsatellites (mono-, di-, tri-, tetra-, penta-, and hexanucleotide repeats) with the following thresholds (unit size, min repeats): ten for mononucleotide, five for dinucleotide, four for trinucleotide, and three repeat units each for tetra-, penta-, and hexanucleotide SSRs. Additionally, the web-based software REPuter (http://bibiserv.techfak.uni-bielefeld.de/reputer/, accessed on 13 December 2023) [23] was employed to analyze repeat sequences. The types of repeats included were forward (F), reverse (R), complement (C), palindromic (P), and tandem, with minimal lengths of 30 bp, maximum lengths of 1000 bp and Hamming distance of 3 bp.

### 2.4. Inverted Repeats Boundary Analysis, Codon Usage Bias, and Genome Comparison

Comparisons were made between the IR/SSC and IR/LSC junctions of six different species by utilizing the online tool IRscope software (https://irScope.shinyapps.io/Irapp/, accessed on 4 December 2023) [24]. An analysis was conducted on the boundaries and identities of the inverted repeat (IR) and single copy (SC) regions for *M. megalocarpa*, as well as for the aforementioned five species. The estimation of codon usage bias was carried out using CodonW v.1.4.2 [25]. RSCU values for each codon were determined based on the coding sequences (CDSs) of the protein-coding genes from *M. megalocarpa* and other species of tribe Megacarpaeeae. Additionally, the comparison of six Brassicaceae chloroplast genomes was performed using the mVISTA [26] program in Shuffle-LAGAN mode, using *M. delavayi* as the reference genome. Results from dynamic visualization helped in observing gene conservation within the chloroplast genomes of the species, as well as identifying the presence or absence of variants and the locations where these variations occur.

### 2.5. Ka/Ks Analysis and Nucleotide Diversity

In order to evaluate the impact of evolutionary selection pressure on the chloroplast genome of the studied species, homologous protein sequences between *M. megalocarpa* and other species of the tribe Megacarpaeeae were obtained using BLASTN. The alignment of shared protein-coding genes was conducted using MAFFT v7.427 [27] software. The Ka/Ks ratios were determined by comparing non-synonymous (Ka) and synonymous (Ks) mutations between *M. megalocarpa* and other species through KaKs_Calculator2.0 [28]. The calculation of nucleotide diversity (Pi values) was performed using DnaSP v.5.10 [29] with a sliding window analysis, where the window size was adjusted to 600 bp, with a step size of 200 bp.

### 2.6. Phylogenetic Analysis and Estimation of Divergence Times

The chloroplast genome sequences of 37 species from nine genera within Brassicaceae were analyzed for phylogenetic reconstruction. Data were sourced from the National Center for Biotechnology Information (NCBI) (Table S1). *Aethionema arabicum* and *Aethionema grandiflorum* were selected to serve as outgroups. Alignment of the sequences was performed using the MAFFT program with default parameters [27]. Subsequently, phylogenetic trees were constructed using two methods: Maximum Likelihood (ML) and Neighbor Joining (NJ). For the ML tree, the GTR+I+G nucleotide replacement model was employed, along with 1000 bootstrap replicates through Phylosuite v.1.2.2 [30]. Visualization of the ML tree was carried out using FigTree v.1.4.2 (download link: http://tree.bio.ed.ac.uk/software/figtree/, accessed on 16 April 2024). As for the NJ tree, the Kimura 2-parameter model was utilized with 1000 bootstrap replicates, analyzed using MEGA v11.0.13 [31].

Estimations for divergence time were conducted using BEAST v 1.8.4 [32] under the uncorrelated lognormal relaxed clock and Yule process model. The Bayesian Markov chain Monte Carlo (MCMC) algorithms were analyzed with other parameter settings, according to Hohmann et al. [33]. Due to the limited macrofossils record of Brassicaceae, three calibration points from the TimeTree database were used: *Ae. arabicum* and other Brassicaceae plants (32–43 Mya), lineages II+ and expanded lineage II, and lineage I (23.4–33.5 Mya), as well as *Lepidium meyenii* and lineage I plants (11.9–20.6 Mya). The MCMC simulation ran for 10,000,000 generations, discarding the initial 10% as burn-in. Visualization of the resulting tree was performed utilizing FigTree.

## 3. Results

### 3.1. Basic Characteristics of the M. megalocarpa Chloroplast Genome

**Figure 2:**
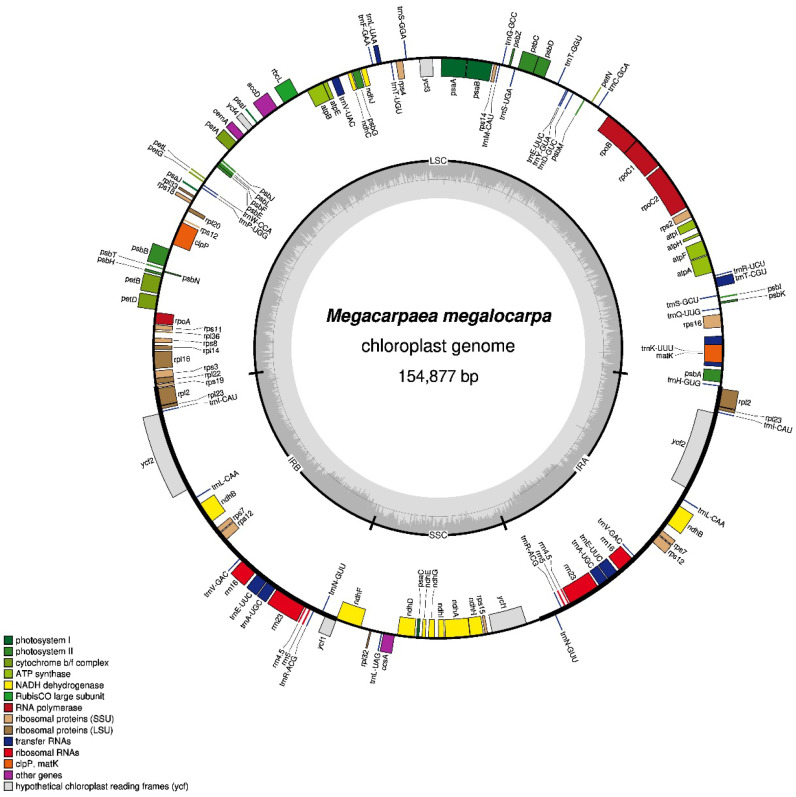
Chloroplast genome map of *M. megalocarpa*. Genes within and outside the circle consist of forward-coding genes and reverse-coding genes. The varying shades of gray in the innermost circle represent the GC and AT contents.

The tetrameric structure of *M. megalocarpa* chloroplast genome measured 154,877 bp, featuring two inverted repeat regions (IR a and IR b), a large single copy (LSC), and a small single copy (SSC), with lengths of 26,446 bp, 84,008 bp, and 17,977 bp, respectively (Figure 2 and Table 1). The overall guanine-cytosine (GC) content stood at 36.20%, with IR a/b showcasing a higher GC content of 42.31% compared to LSC (33.96%) and SSC (29.08%). Despite a variance of 621 bp in the lengths of the chloroplast genomes among the six species, there was minimal fluctuation in their overall GC content and the GC content within the four regions (Table 1).

The chloroplast genome of *M. megalocarpa* contains a total of 129 genes, comprising 85 protein-coding genes, 36 tRNA genes, and 8 rRNA genes. Compared to other species of the tribe Megacarpaeea, *M. megalocarpa* lacks a tRNA, whereas *M. polyandra* and *P. pterocarpum* had two more protein-coding genes, and both contained eight rRNA genes (Table 1). The functional categorization of *M. megalocarpa* chloroplast genome is divided into four categories with 18 groups, which consist of 73 self-replicating genes, 45 photosynthesis-related genes, 5 genes associated with biosynthesis, and 6 genes of unidentified function. Among the identified genes, 18 possess introns. Specifically, six tRNA genes (*trnA-UGC*, *trnE-UUC*, *trnK-UUU*, *trnL-UAA*, *trnT-CGU*, *and trnV-UAC*) and ten protein-coding genes (*ndhA*, *ndhB*, *petB*, *petD*, *atpF*, *rpl16*, *rpl2*, *rps16*, *rpoC1*, *ycf3*) contain a single intron each. Furthermore, the protein-coding genes *clpP* and *rps12* each contain two introns (Table 2). A total of 83 genes (comprising 60 PCGs and 21 tRNA genes) are situated in the LSC region, while 12 genes (including 11 PCGs and 1 tRNA gene) reside in the SSC region. Moreover, 18 genes were duplicated in the IR region, which includes seven protein-coding genes (*ndhB*, *rpl2*, *rpl23*, *rps12*, *rps7*, *ycf1*, *ycf2*), four rRNA genes (*rrn16*, *rrn23*, *rrn4.5*, *rrn5*), and seven tRNA genes (*trnA-UGC*, *trnE-UUC*, *trnI-CAU*, *trnL-CAA*, *trnN-GUU*, *trnR-ACG*, *trnV-GAC*) (Figure 2 and Table 2).

### 3.2. SSRs and Long Repeat Sequences

**Figure 3:**
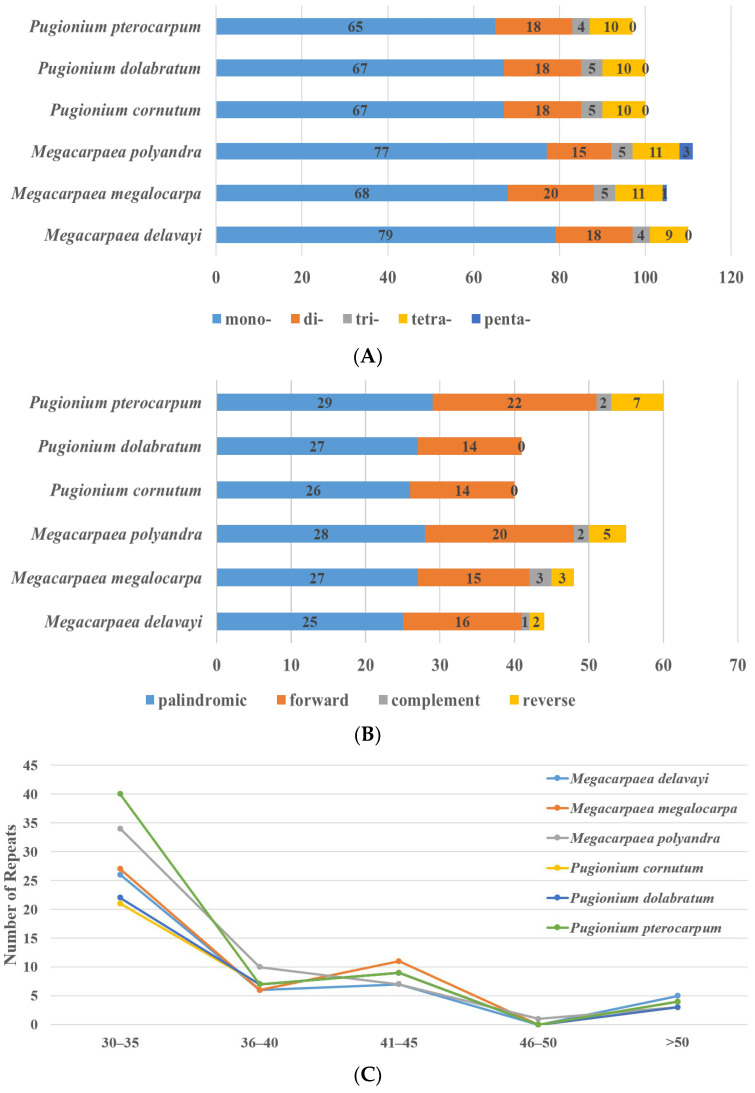
SSRs (simple sequence repeats) and long repetitive sequences in *M. megalocarpa* and other five species. (**A**): types and number of SSRs; (**B**): the quantity of four types of long repetitive sequences; (**C**): length of long repetitive sequences.

The simple sequence repeats (SSRs) generally consisted of DNA sequences containing tandem repeats of one to six bases and were widely distributed at various locations throughout the chloroplast genome. In the genome of *M. megalocarpa*, 105 SSRs were discovered, comprising of 68 mononucleotide repeats, 20 dinucleotide repeats, 5 trinucleotide repeats, 11 tetranucleotide repeats, and 1 pentanucleotide repeat. Pentanucleotide repeats were only present in *M. polyandra* and *M. megalocarpa*, with *M. polyandra* having three and *M. megalocarpa* having one (Figure 3A). Hexanucleotide repeats were absent in six species, with mononucleotide repeats, primarily A/T bases, being the most common, indicating a bias in base composition.

The long repetitive sequences were relatively long DNA sequences that repeat in the chloroplast genome. Within the genome of *M. megalocarpa*, 48 long repetitive sequences were identified, consisting of 27 forward repeats, 15 palindromic repeats, 3 complementary repeats, and 3 reverse repeats. *M. megalocarpa* displayed the highest count of forward and palindromic repeats among the six species, whereas *P. dolabratum* and *P. cornutum* lacked complementary and reverse repeats (Figure 3B). Notably, these sequences were not shorter than 30 bp, as the parameter was set to a minimum value of 30 bp. Mostly 30–35 bp repeats were found in all species studied, with only *M. polyandra* containing one repeat of 46–50 bp in length (Figure 3C).

### 3.3. Expansion and Contraction of the Inverted Repeat Boundaries

**Figure 4:**
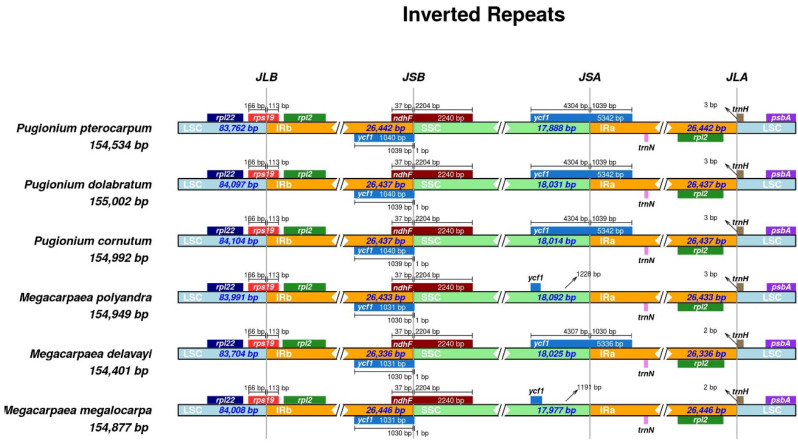
Comparison of the LSC, SSC, and IR region boundaries in the chloroplast genomes of *M. megalocarpa* and other five species. Different boxes represent different gene names. Different colors represent the four regions and gene names.

Differences in genome size among plant species may arise from changes in the IR boundary region of the chloroplast genome. The analysis showed that six species had the same number of *rps19* and *ndhF* genes at the borders of IRb/LSC and IRb/SSC, with gene crossover occurring. The genes *rpl22*, *psbA*, and *trnH* were exclusively located in the LSC region, while the *rpl2* gene in the IR region and the *ycf1* gene in the IRb region did not undergo gene crossover. In contrast to the other four species, the *ycf1* gene was only present in the SSC region in *M. megalocarpa* and *M. polyandra*. Generally, the chosen species displayed a high level of conservation at the boundaries of LSC/IRb, IRb/SSC, and IRa/LSC, whereas the SSC/IRa boundary was more susceptible to mutations (Figure 4).

### 3.4. Codon Usage Bias Analysis

**Figure 5:**
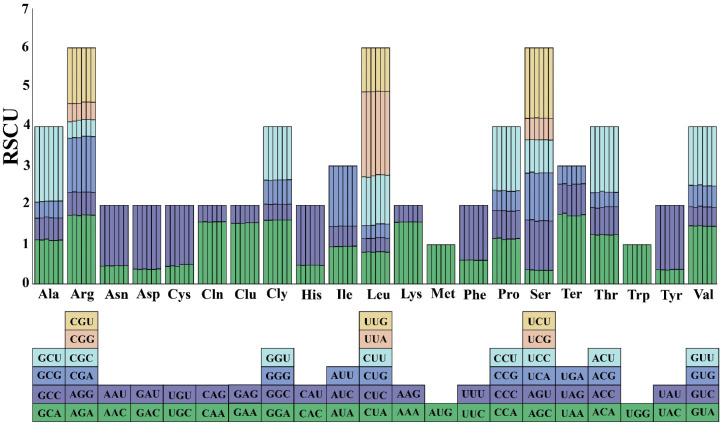
The relative synonymous codon usage of 20 amino acids and the stop codon in the CDS of the chloroplast genome for *M. megalocarpa* and other species of the tribe Megacarpaeeae. The sequence from left to right consists of *M. megalocarpa*, *M. polyandra*, *M. delavayi*, *P. dolabratum*, *P. cornutum*, and *P. pterocarpum*. Different colors represent different codons encoding amino acids.

The analysis of codon usage bias in the chloroplast genome of *M. megalocarpa* revealed almost the same RSCU values among the six species examined. The amino acids with the highest codon diversity across all species were Arginine (Arg), Leucine (Leu), and Serine (Ser), each consisting of six different codons. Conversely, Methionine (Met) and Tryptophan (Trp) were represented by only one codon each. Among the 64 codons studied, 30 exhibited RSCU values exceeding 1, indicating a relatively high frequency of use. Notably, two codons, AUG and UGG, had RSCU values of 1, suggesting no specific bias in their utilization. Additionally, the analysis showed that codons ending in A/U tended to have RSCU values higher than 1, while those ending in C/G had values below 1, which was consistent with the situation in genomes with lower GC content (Figure 5).

### 3.5. Comparative Analysis of Chloroplast Genome Sequences

**Figure 6:**
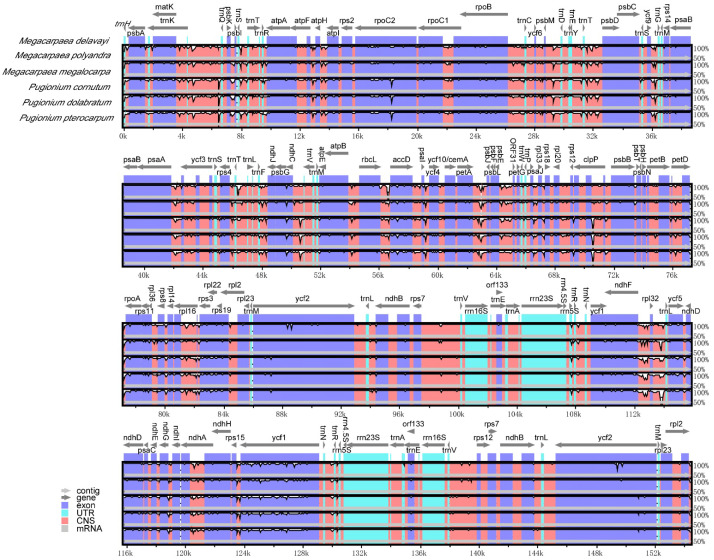
Sequence identity plot comparing the chloroplast genomes of *M. megalocarpa* with those of five other species. Exons, UTR, CNS, and mRNA are marked with different colors. The y-axis represents the percentage of sequence identity from 50% to 100%. Gray arrows above the alignment indicate gene transcription direction. Arrows indicate the annotated genes in the reference genome of *M. delavayi* and their transcription directions.

A comparative analysis of the chloroplast genome sequences from six species indicated no significant rearrangements or inversions in any of the four regions (Figure 6). However, sequence variation was greater in the SSC and LSC regions compared to the IR region. The high conservation of the IR region may be attributed to the conserved properties of the rRNA genes situated within it. In the six species, the coding regions exhibited higher conservation than the non-coding regions. The coding region variants of the gene included *rpoC2*, *psbG*, *accD*, *rpoA*, *rps11*, *rpl22*, *ndhF*, *ycf1*. Meanwhile, variants in the gene’s spacer region were primarily found in regions such as *trnS-trnT*, *atpF-atpH*, *atpH-atpI*, *psbM-trnD*, *trnT-psbD*, *psaA-ycf3*, *trnT-trnL*, *trnF-ndhJ*, *rbcL-accD*, *petA-psbJ*, and *rps15-ycf1*.

### 3.6. Selective Pressure Analyses

**Figure 7:**
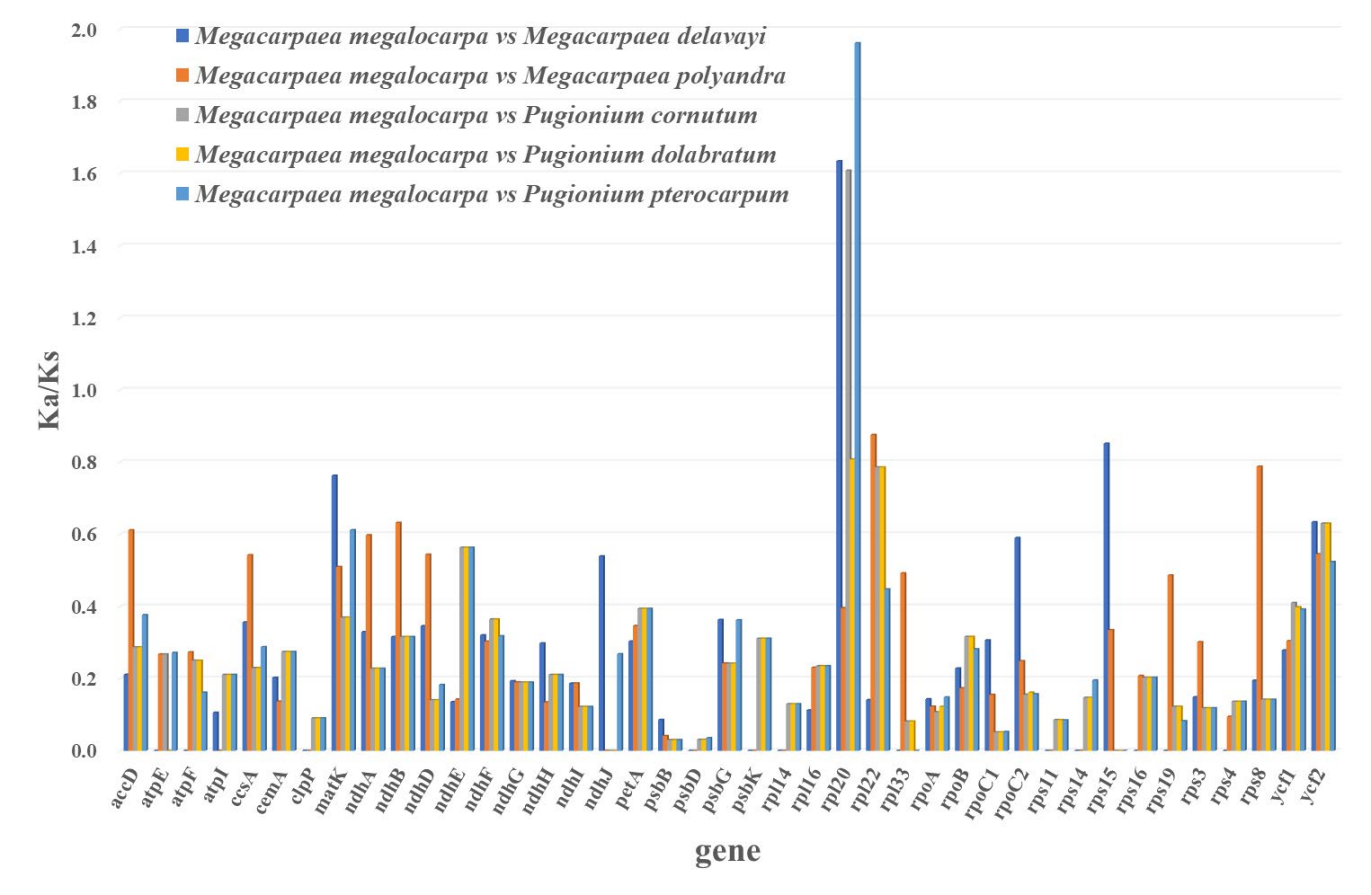
The Ka/Ks values of 41 genes. Ka/Ks values for 41 genes between *M. megalocarpa* and five other species, two-by-two.

Calculating Ka/Ks between species revealed that the Ka/Ks values could not be calculated for some genes (*atpA*, *atpH*, *ndhC*, *petB*, *petG*, *petL*, *petN*, etc.) because Ka or Ks were 0, indicating that these genes were relatively conserved and had no nucleotides to replace. The genes with more than three NA values (Ka tends to infinity) or 0 values (Ks tends to 0) were excluded, and the Ka/Ks values of the remaining 41 genes were analyzed and visualized (Figure 7). The majority of protein-coding genes exhibited Ka/Ks values below one, suggesting that these genes were subject to purifying selection. The Ks/Ks values of *rpl20* were greater than one, indicating that this gene was under positive selection.

### 3.7. Nucleotide Diversity

**Figure 8:**
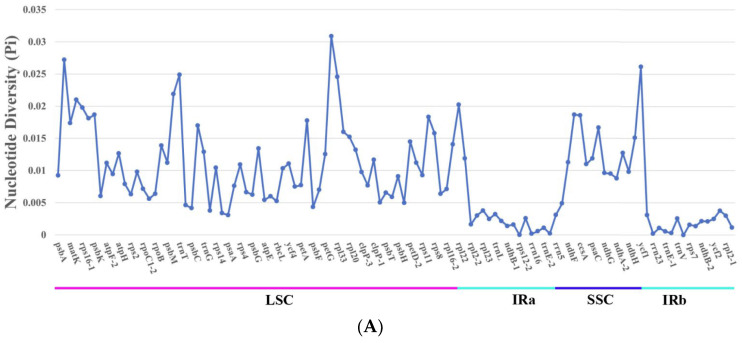

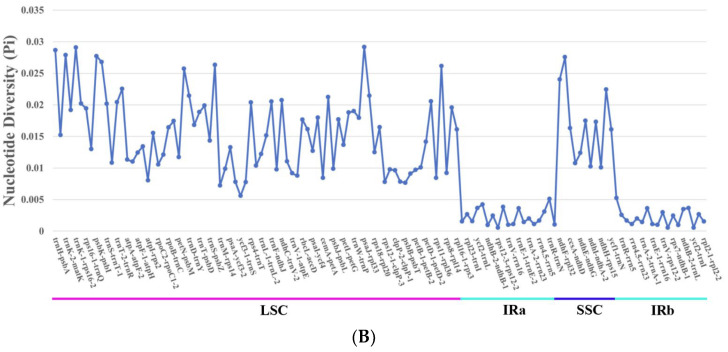
Nucleotide diversity (Pi) of *M. megalocarpa* compared to the other five species. (**A**): protein-coding regions; (**B**): non-coding regions.

Nucleotide diversity values (Pi) of 127 non-coding and 122 coding regions (Tables S2 and S3) revealed that three coding regions (*trnK-2*, *psaJ*, *ycf1*) (Figure 8A) and 10 non-coding regions (*trnH-psbA*, *trnK-1-rps16-2*, *psbK-psbI*, *psbI-trnS*, *psbM-trnD*, *psbZ-trnG*, *psaJ-rpl33*, *rpl36-rps8*, *rpl32-trnL*) (Figure 8B) exhibited Pi values greater than 0.025. Notably, the *psaJ* gene displayed a Pi value exceeding 0.03, signifying variability and diversity at the nucleotide level in this region. The chloroplast genome Pi values ranged from 0 to 0.0308, averaging 0.01041. Among the 13 identified regions, 11 were located in the LSC region, with only two (*rpl32-trnL*, *ycf1*) found in the SSC region. The nucleotide polymorphisms in the IR region were notably lower compared to the LSC and SSC regions, with the SSC region harboring fewer polymorphisms than the LSC region.

### 3.8. Phylogenetic Analyses and Estimation of Divergence Times

**Figure 9:**
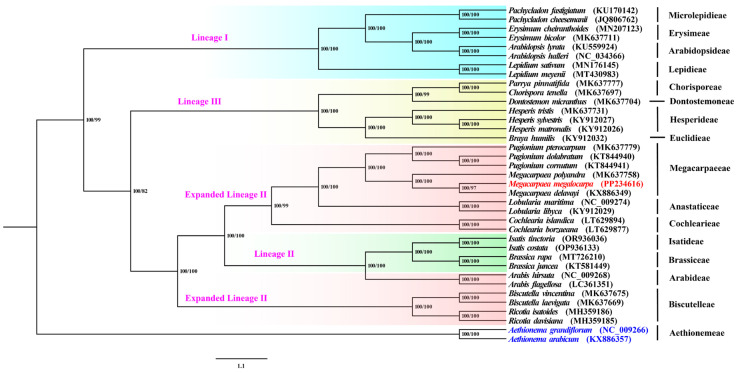
Phylogenetic tree constructed based on 37 species. Numbers at nodes are Maximum Likelihood and Neighbor Joining bootstrap values (BS), separated by “/”. Blue letters represent the outgroups; red letters represent study species; pink letters represent different lineages.

Studies on the taxonomic status and evolutionary relationships of *M. megalocarpa* show that the two phylogenetic methods (ML/NJ) had almost similar topological structures, with generally high support values (Figure 9). Four major lineages were identified: lineages I (Microlepidieae, Erysimeae, Arabidopsideae, Lepidieae), lineages III (Chorisporeae, Dontostemoneaae, Hesperideae, Euclidieae), lineages II (Isatideae, Brassiceae), and expanded lineage II (Megacarpaeeae, Anastaticeae, Cochlearieae, Arabideae, Biscutelleae). The phylogenetic analysis confirmed the phylogenetic position of *M. megalocarpa* in the expanded lineage II of Brassicaceae. The evolutionary trees confirmed that *Megacarpaea* was closely related to *Pugionium*, and that *M. megalocarpa* and *M. delavayi* were the most closely related species, with bootstrap support values higher than 97 for both their ML and NJ trees.

**Figure 10:**
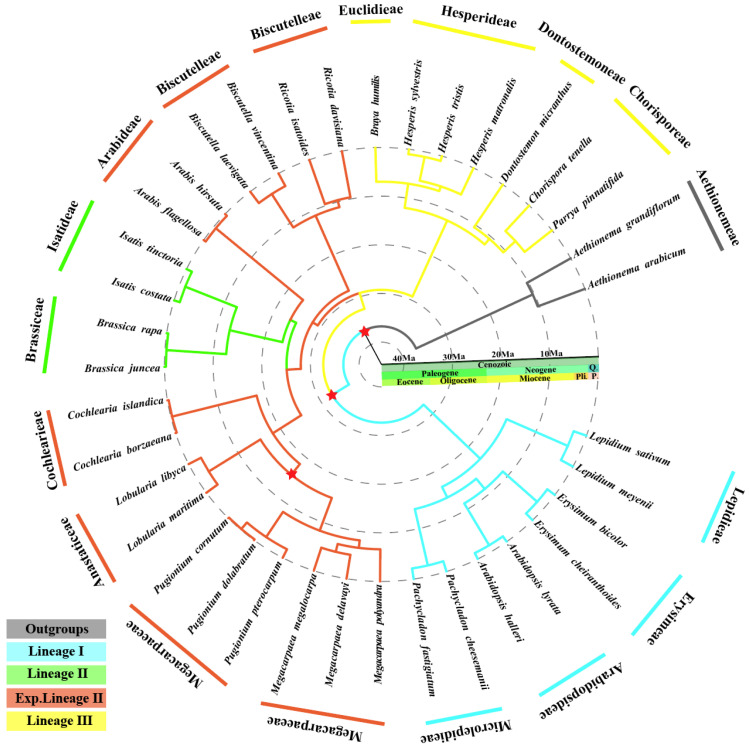
BEAST-derived chronograms of Brassicaceae based on the chloroplast genome sequences with three calibration points (red pentagram) derived from previous studies. Abbreviations of geological time are as follows: Q: Quaternary, P: Pleistocene, Pli: Pliocene.

Estimated divergence times, using phylogenetic relationships as a reference, showed that the core Brassicaceae and Aethionemeae began to split at 36.77 Mya during the Eocene boundary (Figure 10 and Figure S1), while the origins of the major lineages chloroplast genome sequences or clades occurred between the Oligocene and Miocene. The divergences within lineages I and lineages III were dated to Oligocene, while those in the expanded lineage II were estimated to have occurred around 28.02 Mya (23.75–32.76 Mya). The divergence between *Pugionium* and *Megacarpaea* was estimated around 8.18 Mya (5.77–12.50 Mya). *M. megalocarpa* diverged from *M. delavayi* about 4.97 Mya (2.82–6.76 Mya), whereas *M. megalocarpa* diverged from *M. polyandra* about 6.63 Mya (3.65–8.54 Mya).

## 4. Discussion

### 4.1. Architecture of Chloroplast Genomes in Megacarpaeeae

In this study, we present the first assembly and annotation of the *M. megalocarpa* whole chloroplast genome. Five previously reported closely related species were used in a basic comparative study to confirm the close relationships found within the genus *Megacarpaea* and with other genera. The size, structure, and gene content of the chloroplast genome in this species were highly similar to those of *P. dolabratum*, *P. cornutum* [34], and other Brassicaceae species [35,36], indicating a high conservation of the chloroplast genome structure. The guanine and cytosine (GC) content in the IR a/b region of *M. megalocarpa* was found to be higher than that in the LSC and SSC regions, which is consistent with previous chloroplast genome studies in species such as *Sinapis alba* and *Eutrema japonicum* [37,38]. This suggests a high conservation of the IR region, possibly due to the conserved nature of the rRNA genes located in this region, resulting in a higher GC content in the IR region than in other regions. Moreover, the GC content exhibited variation across different species, which has influenced the distribution, environmental adaptability, and lifestyles of species [39].

Simple sequence repeats (SSRs) can be extensive applications across various biological fields, such as genetic map development and crop improvement, and is an important tool applied in genetic relationships, population structure, and phylogenetic analysis among species [40,41]. The SSRs in the genomes of the six species primarily consisted of single nucleotide repeats. These repeats showed a bias towards A/T base compositions, which could be attributed to the higher susceptibility of A/T to change compared to G/C. This bias may be linked to the evolutionary history of the species or their environmental adaptations [42]. Among the species, only *M. polyandra* and *M. megalocarpa* possessed three and one pentanucleotide repeat sequences, respectively. These variants were valuable for identifying polymorphic regions at the individual level and serve as specific markers for genetic diversity analysis [43]. In this study, most of the long repetitive sequences were forward (F) and palindromic (P) repeats, which have also been observed in other studies of angiosperms [35,44,45]. This further suggests that forward and palindromic repeat sequences play an important role in maintaining structural and functional stability in the genome and contribute to maintaining the integrity and stability of the genome [46]. Additionally, the analysis of long repetitive sequence length showed a gradual decrease in the number of repetitive sequences as the sequence length increased, a phenomenon that was also found in the study of *Stemona parviflora* [47].

The differences in chloroplast genome length and structure were attributed to the expansion and contraction of inverted repeat (IR) boundaries [48]. The contraction of the *ycf1* gene at the *M. megalocarpa* and *M. polyandra* SSC/IRa boundaries was observed, and this variability has also been observed in other species, such as *Rheum*, *Quercus*, and *Camellia* [49,50,51]. This variability is attributed to the high variability and susceptibility to mutation of the *ycf1* gene, which has multiple mutation sites, and it encodes a protein that is a component of the chloroplast inner envelope membrane protein translocon [50,52]. However, further validation was required to confirm the potential of the highly polymorphic *ycf1* gene as a core DNA barcode [53]. The boundaries between LSC/IRb (JLB), SSC/IRb (JSB), and LSC/IRa (JLA) were identical, indicating a closer relationship, which has been supported by subsequent phylogenetic studies. Additionally, significant differences in codon usage between species were observed [54]. The amino acids with the highest variety of codon usage in this study were Arg, Leu, and Ser, while Met and Trp had only one codon. These findings were consistent with the results observed in *S. parviflora* and *Cyathula officinalis* [47,55]. Among all the codons studied, Leu with the codon UUA exhibits the highest usage bias, while Leu with the codon CUG exhibits the lowest usage bias. Codons AUG and UGG show no usage preference (Table S4). A comparison revealed that almost all codons ending in A/U have RSCU values greater than one, while those ending in C/G have RSCU values less than one. This phenomenon might be attributed to the higher content of A and T bases, resulting in an obvious bias for A or T termination codons, a preference that may arise from evolutionary pressures and genetic alterations [56].

The inheritance pattern of the chloroplast genome was matrilineal, with a relatively low incidence of base substitutions and genome structure rearrangement events [57]. This was supported by the absence of gene rearrangements and inversions in *M. megalocarpa* and other closely related species. While there were differences in the mVISTA analyses of the *M. megalocarpa* chloroplast genome, most of these differences were found in the intergenic spacer regions, with overall conservation remaining consistent. Not only do Brassicaceae plants exhibit this phenomenon, but Orchidaceae and Betulaceae also show similar results, supporting the conservation of the chloroplast genome [58,59]. The ratio of non-synonymous-to-synonymous mutations (Ka/Ks) in genes encoding proteins was an important indicator of selection pressure in molecular evolution [60]. In this study, only the *rpl20* gene showed evidence of positive selection, was associated with transcription and translation, and suggested its involvement in adaptive evolution and environmental adaptation [61,62]. The Ka/Ks values of the *rpl20* gene showed positive selection in *M. delavayi*, *P. cornutum*, and *P. pterocarpum*, but purifying selection in *M. megalocarpa*, *M. polyandra*, and *P. dolabratum*, indicating that different species experienced varying evolutionary pressures on this gene. Nucleotide diversity studies have shown that the IR region of the species was less polymorphic, compared to the LSC and SSC regions. This was attributed to the lower variability of the conserved rRNA genes in the IR region [63]. The 13 regions with high Pi values (>0.025) were more susceptible to nucleotide substitutions during evolution. Notably, the *psaJ* gene (Pi > 0.03) in the LSC region could serve as an effective molecular marker for species identification, providing valuable data and phylogenetic information for genetic evolutionary analyses.

### 4.2. Phylogeny of Chloroplast Genome of Megacarpaeeae

In phylogenetic trees, the ML and NJ trees constructed exhibited similar topologies. However, the lack of a robust, densely sampled Brassicaceae Tree of Life has resulted in a variety of different phylogenetic relationships at present. A system of four major lineages, as opposed to a system of three [64,65] or five [2,9] major lineage divisions, provides a good indication of the phylogenetic position of Megacarpaeeae. Therefore, four major lineages were identified, as well as the phylogenetic position of *M. megalocarpa* in the expanded lineage II of Brassicaceae, which is consistent with the findings of Kiefer et al. [13]. Notably, *Megacarpaea* and *Pugionium* formed a highly supported monophyletic taxon, which is consistent with previous studies [7,37,64]. This finding further confirms the close relationship between the *Megacarpaea* and *Pugionium* species. Additionally, *M. delavayi* was found to be more closely related to *M. megalocarp* than to *M. polyandra*.

The ages of the major Brassicaceae splits are in agreement with previously published results; most Brassicaceae species mainly diverged in the middle Miocene to Pleistocene [7]. The divergences within *M. megalocarpa* with *M. delavayi* and *M. polyandra* were estimated to have occurred around 6.63 Mya (3.65 Mya–8.54 Mya), which was basically consistent with the time-differentiation results of previous analyses, based on the chloroplast genomes of *M. delavayi* and *M. polyandra* [9]. *M. megalocarpa* and *M. delavayi* diverged separately about 4.97 Mya (2.82 Mya–6.76 Mya). This might have been due to the rapid uplift of the Tibetan Plateau in the Neogene, resulting in the formation of mountains such as the Tian Mountains and Qilian Mountains, and a harsh drought in the northwest from the late Miocene to Pliocene, leading to the differentiation of *M. megalocarpa* [66,67]. *M. megalocarpa* grows in the sandy deserts and alkaline plains of northwestern China (Qinghai and Xinjiang). *M. delavayi* grows in the swampy meadows, on the steep grassy slopes, and in the open thickets of southwest China (Gansu, Qinghai, Sichuan, Xizang, Yunnan) at elevations of 3300–4800 m [10]. Accordingly, *M. megalocarpa*’s and *M. delavayi*’s divergence could be inferred to be possibly related to violent geological movements from the massive Tibetan Plateau uplift, as well as the aridification of the northwestern region [67,68].

## 5. Conclusions

In this study, the chloroplast genome of *M. megalocarpa* was assembled and characterized and compared with other species of the tribe Megacarpaeeae. The results confirmed the previously proposed hypothesis that the chloroplast genome (154,877 bp) of *M. megalocarpa* shares similarities with the *Megacarpaea* species in terms of characteristics. It is worth noting that the *psaJ* gene in the LSC region can be used as a molecular marker for species identification. Phylogenetic analysis confirmed that *M. megalocarpa* and *M. delavayi* are closely related and differentiated independently around 4.97 Ma, suggesting that this may be related to the violent geological movement associated with the large-scale uplift of the Tibetan Plateau. This provides valuable genetic resources for understanding phylogenetic relationships within the genus and refining the complex classification and species identification of Brassicaceae plants.

## Figures and Tables

**Table 1:** Chloroplast genome information of *M. megalocarpa* and other species of the tribe Megacarpaeeae.

	Length (bp)				GC Content (%)				Number of Gene			
	Genome	LSC	IR	SSC	Total	LSC	IR	SSC	Total	Protein Coding	tRNA	rRNA
*M. megalocarpa*	154,877	84,008	26,446	17,977	36.20	33.96	42.31	29.08	129	85	36	8
*M. delavayi*	154,401	83,704	26,336	18,025	36.20	33.89	42.34	28.98	130	85	37	8
*M. polyandra*	154,949	83,991	26,433	18,092	36.10	33.85	42.28	28.91	132	87	37	8
*P. cornutum*	154,992	84,104	26,437	18,014	36.20	33.92	42.36	29.05	130	85	37	8
*P. dolabratum*	155,022	84,097	26,437	18,031	36.20	33.93	42.36	29.03	130	85	37	8
*P. pterocarpum*	154,534	83,762	26,442	17,888	36.30	33.92	42.36	29.16	132	87	37	8

**Table 2:** Functional classification of the chloroplast genome of *M. megalocarpa*. Note: gene*: gene with one intron; gene**: gene with two introns; gene (2): number of copies of multi-copy genes.

Category	Gene Group	Gene Name
Photosynthesis	Subunits of photosystem I	*psaA*, *psaB*, *psaC*, *psaI*, *psaJ*
	Subunits of photosystem II	*psbA*, *psbB*, *psbC*, *psbD*, *psbE*, *psbF*, *psbG*, *psbH*, *psbI*, *psbJ*, *psbK*, *psbL*, *psbM*, *psbN*, *psbT*, *psbZ*
	Subunits of NADH dehydrogenase	ndhA*, ndhB*(2), *ndhC*, *ndhD*, *ndhE*, *ndhF*, *ndhG*, *ndhH*, *ndhI*, *ndhJ*
	Subunits of cytochrome b/f complex	*petA*, petB*, petD*, *petG*, *petL*, *petN*
	Subunits of ATP synthase	*atpA*, *atpB*, *atpE*, atpF*, *atpH*, *atpI*
	Large subunit of rubisco	*rbcL*
Self-replication	Proteins of large ribosomal subunit	*rbcL*
	Proteins of small ribosomal subunit	*rps11*, rps12**(2), *rps14*, *rps15*, rps16*, *rps18*, *rps19*, *rps2*, *rps3*, *rps4*, *rps7(2)*, *rps8*
	Subunits of RNA polymerase	*rpoA*, *rpoB*, rpoC1*, *rpoC2*
	Ribosomal RNAs	*rrn16(2)*, *rrn23(2)*, *rrn4.5(2)*, *rrn5(2)*
	Transfer RNAs	trnA-UGC*(2), *trnC-GCA*, *trnD-GUC*, *trnE-UUC*, trnE-UUC*(2), *trnF-GAA*, *trnG-GCC*, *trnH-GUG*, *trnI-CAU(2)*, trnK-UUU*, *trnL-CAA(2)*, trnL-UAA*, *trnL-UAG*, *trnM-CAU*, *trnN-GUU(2)*, *trnP-UGG*, *trnQ-UUG*, *trnR-ACG(2)*, *trnR-UCU*, *trnS-GCU*, *trnS-GGA*, *trnS-UGA*, trnT-CGU*, *trnT-GGU*, *trnT-UGU*, *trnV-GAC(2)*, trnV-UAC*, *trnW-CCA*, *trnY-GUA*
Other genes	Maturase	*matK*
	Protease	clpP**
	Envelope membrane protein	*cemA*
	Acetyl-CoA carboxylase	*accD*
	c-type cytochrome synthesis gene	*ccsA*
Genes of unknown function	Conserved hypothetical chloroplast ORF	*ycf1(2)*, *ycf2(2)*, ycf3*, *ycf4*

## Data Availability

The chloroplast genome sequences that support the findings of this study are openly available in NCBI, GenBank accession number: PP234616. The raw sequencing data for this study are publicly available in the NCBI Sequence Read Archive (SRA), the BioProject number: PRJNA1129693.

## Supplementary Materials

The following supporting information can be downloaded at: https://www.mdpi.com/article/10.3390/genes15070886/s1. Table S1: GenBank IDs for chloroplast genome sequences of 30 species and *M. megalocarpa*. Table S2: Nucleotide diversity (Pi) values were calculated for 127 non-coding. Table S3: Nucleotide diversity (Pi) values were calculated for 122 coding regions. Table S4: RSCU values of the chloroplast genome for *M. megalocarpa* and other species of tribe Megacarpaeeae. Figure S1: Divergence time (million years ago) of Brassicaceae species inferred from the complete chloroplast genome data.

## References

[1] Al-Shehbaz I.A. (2012). A Generic and Tribal Synopsis of the Brassicaceae (Cruciferae). Taxon.

[2] Nikolov L.A., Shushkov P., Nevado B., Gan X., Al-Shehbaz I.A., Filatov D., Bailey C.D., Tsiantis M. (2019). Resolving the Backbone of the Brassicaceae Phylogeny for Investigating Trait Diversity. New Phytol..

[3] Shankar S., Segaran G., Sundar R.D.V., Settu S., Sathiavelu M. (2019). Brassicaceae—A Classical Review on Its Pharmacological Activities. Int. J. Pharm. Sci. Rev. Res..

[4] Higdon J., Delage B., Williams D., Dashwood R. (2007). Cruciferous Vegetables and Human Cancer Risk: Epidemiologic Evidence and Mechanistic Basis. Pharmacol. Res..

[5] Murillo G., Mehta R.G. (2001). Cruciferous Vegetables and Cancer Prevention. Nutr. Cancer.

[6] Ilahy R., Tlili I., Pék Z., Montefusco A., Siddiqui M.W., Homa F., Hdider C., R’Him T., Lajos H., Lenucci M.S. (2020). Pre-and Post-Harvest Factors Affecting Glucosinolate Content in Broccoli. Front. Nutr..

[7] Guo X., Liu J., Hao G., Zhang L., Mao K., Wang X., Zhang D., Ma T., Hu Q., Al-Shehbaz I.A. (2017). Plastome Phylogeny and Early Diversification of Brassicaceae. BMC Genom..

[8] Huang C.-H., Sun R., Hu Y., Zeng L., Zhang N., Cai L., Zhang Q., Koch M.A., Al-Shehbaz I., Edger P.P. (2016). Resolution of Brassicaceae Phylogeny Using Nuclear Genes Uncovers Nested Radiations and Supports Convergent Morphological Evolution. Mol. Biol. Evol..

[9] Hendriks K.P., Kiefer C., Al-Shehbaz I.A., Bailey C.D., Van Huysduynen A.H., Nikolov L.A., Nauheimer L., Zuntini A.R., German D.A., Franzke A. (2023). Global Brassicaceae Phylogeny Based on Filtering of 1,000-Gene Dataset. Curr. Biol..

[10] Cheo T., Lu L., Yang G., Al-Shehbaz I., Dorofeev V. (2001). Flora of China.

[11] Shen L., Liu X., Shi G., Yang Y., Li B. (2009). Effect of *Megacarpaea delavayi* Franch on Digestive Juice in Rat with Heat Due to Food Stagnation. Chin. J. Ethnomed. Ethnopharm..

[12] Singh A., Nautiyal M.C., Curti R.N., Fenu G. (2022). The Phenological Growth Stages of *Megacarpaea polyandra* Benth. Ex Madden: A High Valued Traditional Medicinal Plant of the Himalaya. Genet. Resour. Crop Evol..

[13] Kiefer M., Schmickl R., German D.A., Mandáková T., Lysak M.A., Al-Shehbaz I.A., Franzke A., Mummenhoff K., Stamatakis A., Koch M.A. (2014). BrassiBase: Introduction to a Novel Knowledge Database on Brassicaceae Evolution. Plant Cell Physiol..

[14] Ahmed I. (2015). Chloroplast Genome Sequencing: Some Reflections. Next Gen. Seq. Appl..

[15] Wu Y., Liu F., Yang D.-G., Li W., Zhou X.-J., Pei X.-Y., Liu Y.-G., He K.-L., Zhang W.-S., Ren Z.-Y. (2018). Comparative Chloroplast Genomics of Gossypium Species: Insights into Repeat Sequence Variations and Phylogeny. Front. Plant Sci..

[16] Wang J., He W., Xiang K., Wu Z., Gu C. (2023). Advancements in Plant Phylogenomics in the Genomic Era. J. Zhejiang AF Univ..

[17] Bolger A.M., Lohse M., Usadel B. (2014). Trimmomatic: A Flexible Trimmer for Illumina Sequence Data. Bioinformatics.

[18] Jin J.-J., Yu W.-B., Yang J.-B., Song Y., de Pamphilis C.W., Yi T.-S., Li D.-Z. (2020). GetOrganelle: A Fast and Versatile Toolkit for Accurate de Novo Assembly of Organelle Genomes. Genome Biol..

[19] Shi L., Chen H., Jiang M., Wang L., Wu X., Huang L., Liu C. (2019). CPGAVAS2, an Integrated Plastome Sequence Annotator and Analyzer. Nucleic Acids Res..

[20] Kearse M., Moir R., Wilson A., Stones-Havas S., Cheung M., Sturrock S., Buxton S., Cooper A., Markowitz S., Duran C. (2012). Geneious Basic: An Integrated and Extendable Desktop Software Platform for the Organization and Analysis of Sequence Data. Bioinformatics.

[21] Lohse M., Drechsel O., Kahlau S., Bock R. (2013). OrganellarGenomeDRAW—A Suite of Tools for Generating Physical Maps of Plastid and Mitochondrial Genomes and Visualizing Expression Data Sets. Nucleic Acids Res..

[22] Thiel T., Michalek W., Varshney R., Graner A. (2003). Exploiting EST Databases for the Development and Characterization of Gene-Derived SSR-Markers in Barley (*Hordeum vulgare* L.). Theor. Appl. Genet..

[23] Kurtz S. (2001). REPuter: The Manifold Applications of Repeat Analysis on a Genomic Scale. Nucleic Acids Res..

[24] Amiryousefi A., Hyvönen J., Poczai P. (2018). IRscope: An Online Program to Visualize the Junction Sites of Chloroplast Genomes. Bioinformatics.

[25] Peden J. (2000). Analysis of Codon Usage. Ph.D. Thesis.

[26] Frazer K.A., Pachter L., Poliakov A., Rubin E.M., Dubchak I. (2004). VISTA: Computational Tools for Comparative Genomics. Nucleic Acids Res..

[27] Katoh K., Rozewicki J., Yamada K.D. (2019). MAFFT Online Service: Multiple Sequence Alignment, Interactive Sequence Choice and Visualization. Brief. Bioinf..

[28] Wang D., Zhang Y., Zhang Z., Zhu J., Yu J. (2010). KaKs_Calculator 2.0: A Toolkit Incorporating γ-Series Methods and Sliding Window Strategies. Genom. Proteom. Bioinform..

[29] Librado P., Rozas J. (2009). DnaSP v5: A Software for Comprehensive Analysis of DNA Polymorphism Data. Bioinformatics.

[30] Zhang D., Gao F., Jakovlić I., Zou H., Zhang J., Li W.X., Wang G.T. (2020). PhyloSuite: An Integrated and Scalable Desktop Platform for Streamlined Molecular Sequence Data Management and Evolutionary Phylogenetics Studies. Mol. Ecol. Resour..

[31] Tamura K., Stecher G., Kumar S. (2021). MEGA11: Molecular Evolutionary Genetics Analysis Version 11. Mol. Biol. Evol..

[32] Drummond A.J., Suchard M.A., Xie D., Rambaut A. (2012). Bayesian Phylogenetics with BEAUti and the BEAST 1.7. Mol. Biol. Evol..

[33] Hohmann N., Wolf E.M., Lysak M.A., Koch M.A. (2015). A Time-Calibrated Road Map of Brassicaceae Species Radiation and Evolutionary History. Plant Cell.

[34] Hu Q., Hu H., Guo X., Ma Y., Liu J., Ma T. (2016). Characterization of the Complete Chloroplast Genome of Two Sister Species of *Pugionium* (Brassicaceae). Conserv. Genet. Resour..

[35] Javaid N., Ramzan M., Khan I.A., Alahmadi T.A., Datta R., Fahad S., Danish S. (2022). The Chloroplast Genome of *Farsetia hamiltonii* Royle, Phylogenetic Analysis, and Comparative Study with Other Members of Clade C of Brassicaceae. BMC Plant Biol..

[36] Shang S., Zhao L., Xu T., Li C., Shen R. (2021). The Complete Chloroplast Genome of Lepidium *Latifolium linnaeus* and Phylogenetic Analysis of Brassicaceae. Mitochondrial DNA B.

[37] Du X., Zeng T., Feng Q., Hu L., Luo X., Weng Q., He J., Zhu B. (2020). The Complete Chloroplast Genome Sequence of Yellow Mustard (*Sinapis alba* L.) and Its Phylogenetic Relationship to Other Brassicaceae Species. Gene.

[38] Li M., Zhang R., Li J., Zheng K., Xiao J., Zheng Y. (2021). Analyses of Chloroplast Genome of *Eutrema japonicum* Provide New Insights into the Evolution of Eutrema Species. Agronomy.

[39] Mann S., Chen Y.-P.P. (2010). Bacterial Genomic G+C Composition-Eliciting Environmental Adaptation. Genomics.

[40] Ebert D., Peakall R. (2009). Chloroplast Simple Sequence Repeats (cpSSRs): Technical Resources and Recommendations for Expanding cpSSR Discovery and Applications to a Wide Array of Plant Species. Mol. Ecol. Resour..

[41] George B., Bhatt B.S., Awasthi M., George B., Singh A.K. (2015). Comparative Analysis of Microsatellites in Chloroplast Genomes of Lower and Higher Plants. Curr. Genet..

[42] Kuang D.-Y., Wu H., Wang Y.-L., Gao L.-M., Zhang S.-Z., Lu L. (2011). Complete Chloroplast Genome Sequence *of Magnolia kwangsiensis* (Magnoliaceae): Implication for DNA Barcoding and Population Genetics. Genome.

[43] Powell W., Morgante M., McDevitt R., Vendramin G.G., Rafalski J.A. (1995). Polymorphic Simple Sequence Repeat Regions in Chloroplast Genomes: Applications to the Population Genetics of Pines. Proc. Natl. Acad. Sci. USA.

[44] Sun J., Wang Y., Liu Y., Xu C., Yuan Q., Guo L., Huang L. (2020). Evolutionary and Phylogenetic Aspects of the Chloroplast Genome of Chaenomeles Species. Sci. Rep..

[45] Zhang Y., Du L., Liu A., Chen J., Wu L., Hu W., Zhang W., Kim K., Lee S.-C., Yang T.-J. (2016). The Complete Chloroplast Genome Sequences of Five Epimedium Species: Lights into Phylogenetic and Taxonomic Analyses. Front. Plant Sci..

[46] Wei S., Liufu Y., Zheng H., Chen H., Lai Y., Liu Y., Ye Q., Tang S. (2023). Using Phylogenomics to Untangle the Taxonomic Incongruence of Yellow-flowered *Camellia* Species (Theaceae) in China. J. Syst. Evol..

[47] Wei R., Li Q. (2022). The Complete Chloroplast Genome of Endangered Species *Stemona parviflora*: Insight into the Phylogenetic Relationship and Conservation Implications. Genes.

[48] Wang W., Messing J. (2011). High-Throughput Sequencing of Three Lemnoideae (Duckweeds) Chloroplast Genomes from Total DNA. PLoS ONE.

[49] Yang J.-B., Yang S.-X., Li H.-T., Yang J., Li D.-Z. (2013). Comparative Chloroplast Genomes of *Camellia* Species. PLoS ONE.

[50] Yang Y., Zhou T., Duan D., Yang J., Feng L., Zhao G. (2016). Comparative Analysis of the Complete Chloroplast Genomes of Five *Quercus* Species. Front. Plant Sci..

[51] Zhou T., Zhu H., Wang J., Xu Y., Xu F., Wang X. (2020). Complete Chloroplast Genome Sequence Determination of *Rheum* Species and Comparative Chloroplast Genomics for the Members of Rumiceae. Plant Cell Rep..

[52] Kikuchi S., Bédard J., Hirano M., Hirabayashi Y., Oishi M., Imai M., Takase M., Ide T., Nakai M. (2013). Uncovering the Protein Translocon at the Chloroplast Inner Envelope Membrane. Science.

[53] Li L., Hu Y., He M., Zhang B., Wu W., Cai P., Huo D., Hong Y. (2021). Comparative Chloroplast Genomes: Insights into the Evolution of the Chloroplast Genome of *Camellia sinensis* and the Phylogeny of *Camellia*. BMC Genom..

[54] Gouy M., Gautier C. (1982). Codon Usage in Bacteria: Correlation with Gene Expressivity. Nucleic Acids Res..

[55] Guo H., Wang L., Xu W., Huo Z., Yang P., Zhang Q., Wang H., Li P., Lu X. (2022). The Complete Chloroplast Genome Sequence of *Cyathula officinalis* and Comparative Analysis with Four Related Species. Gene.

[56] Gao Y., Chen Z., Li X., Malik K., Li C. (2024). Comparative Analyses of Complete Chloroplast Genomes of *Microula sikkimensis* and Related Species of Boraginaceae. Genes.

[57] Qian F., Gao Z., Hu L., Wang H. (2022). Characteristics of the Chloroplast Genome and Phylogenetic Studies of *Crambe abyssinica*. Biotechnol. Bull..

[58] Dong W.-L., Wang R.-N., Zhang N.-Y., Fan W.-B., Fang M.-F., Li Z.-H. (2018). Molecular Evolution of Chloroplast Genomes of Orchid Species: Insights into Phylogenetic Relationship and Adaptive Evolution. Int. J. Mol. Sci..

[59] Hu G., Cheng L., Huang W., Cao Q., Zhou L., Jia W., Lan Y. (2020). Chloroplast Genomes of Seven Species of Coryloideae (Betulaceae): Structures and Comparative Analysis. Genome.

[60] Dos Reis M. (2015). How to Calculate the Non-Synonymous to Synonymous Rate Ratio of Protein-Coding Genes under the Fisher-Wright Mutation-Selection Framework. Biol. Lett..

[61] Yang Z., Nielsen R. (2000). Estimating Synonymous and Nonsynonymous Substitution Rates under Realistic Evolutionary Models. Mol. Biol. Evol..

[62] Yu T., Gao J., Liao P.-C., Li J.-Q., Ma W.-B. (2022). Insights into Comparative Analyses and Phylogenomic Implications of *Acer* (Sapindaceae) Inferred from Complete Chloroplast Genomes. Front. Genet..

[63] Jansen R.K., Raubeson L.A., Boore J.L., de Pamphilis C.W., Chumley T.W., Haberle R.C., Wyman S.K., Alverson A.J., Peery R., Herman S.J. (2005). Methods for Obtaining and Analyzing Whole Chloroplast Genome Sequences. Methods in Enzymology.

[64] Liu L., Du X., Guo C., Li D. (2021). Resolving Robust Phylogenetic Relationships of Core Brassicaceae Using Genome Skimming Data. J. Syst. Evol..

[65] Couvreur T.L.P., Franzke A., Al-Shehbaz I.A., Bakker F.T., Koch M.A., Mummenhoff K. (2010). Molecular Phylogenetics, Temporal Diversification, and Principles of Evolution in the Mustard Family (Brassicaceae). Mol. Biol. Evol..

[66] Sun J., Liu T. (2006). The Age of the Taklimakan Desert. Science.

[67] Dai S., Zhang M., Peng D., Wang H., Wu M., Chen R. (2013). Tectonic and Climatic Pattern Evolution of the Middle-Cenozoic in Northwest China. Mar. Geol. Quat. Geol..

[68] Li X., Dong G. (2006). Discussion on the Formation Era and Causes of Arid Environment in Northwest China. Quat. Sci..

