# Supplementary material for: Complete Chloroplast Genome of Megacarpaea megalocarpa and Comparative Analysis with Related Species from Brassicaceae
Source: Genes (Basel). 2024 Jul 5;15(7):886. doi: 10.3390/genes15070886 (PMC11276580; doi:10.3390/genes15070886)
Supplement: Supplementary file 1 [file genes-15-00886-s001.zip › genes-3072957-supplementary.pdf]

genes

MDPI

Article

Complete Chloroplast Genome of *Megacarpaea megalocarpa* and Comparative Analysis with Related Species from Brassicaceae

Zhuo Zhang, Xiaojun Shi, Haowen Tian, Juan Qiu, Hanze Ma and Dunyan Tan

Table S1. GenBank IDs for cp genome sequences of 37 species and *Megacarpaea megalocarpa*.

GenBank ID	species	GenBank ID	species
MT430983	*Lepidium meyenii*	MK637758	*Megacarpaea polyandra*
MN176145	*Lepidium sativum*	KX886349	*Megacarpaea delavayi*
KU170142	*Pachycladon fastigiatum*	PP234616	*Megacarpaea megalocarpa*
JQ806762	*Pachycladon cheesemanii*	KT844941	*Pugionium cornutum*
KU559924	*Arabidopsis lyrata*	KT844940	*Pugionium dolabratum*
NC_034366	*Arabidopsis halleri*	MK637779	*Pugionium pterocarpum*
MN207123	*Erysimum cheiranthoides*	LT629877	*Cochlearia borzaeana*
MK637711	*Erysimum bicolor*	LT629894	*Cochlearia islandica*
MK637704	*Dontostemon micranthus*	LC361351	*Arabis flagellosa*
MK637697	*Chorispora tenella*	NC_009268	*Arabis hirsuta*
KY912026	*Hesperis matronalis*	MH359186	*Ricotia isatoides*
KY912027	*Hesperis sylvestris*	MH359185	*Ricotia davisiana*
MK637731	*Hesperis tristis*	NC_009274	*Lobularia maritima*
MK637777	*Parrya pinnatifida*	KY912029	*Lobularia libyca*
KY912032	*Braya humilis*	MK637675	*Biscutella vincentina*
KT581449	*Brassica juncea*	MK637669	*Biscutella laevigata*
MT726210	*Brassica rapa*	KX886357	*Aethionema arabicum*
OP936133	*Isatis costata*	NC_009266	*Aethionema grandiflorum*
OR936036	*Isatis tinctoria*		

Table S2. Nucleotide diversity (Pi) values were calculated for 127 non-coding.

non-coding regions	Pi	non-coding regions	Pi	non-coding regions	Pi
trnH-psbA	0.0287	psbG-ndhC	0.0098	rps12-3-rps12-2	0.0006
psbA-trnK-2	0.0152	ndhC-trnV-2	0.0207	rps12-2-trnV	0.0038
trnK-2-matK	0.0279	trnV-2-trnV-1	0.0110	trnV-rrn16	0.0010
matK-trnK-1	0.0192	trnV-1-atpE	0.0092	rrn16-trnE-1	0.0011
trnK-1-rps16-2	0.0291	atpB-rbcL	0.0088	trnE-1-trnE-2	0.0036
rps16-2-rps16-1	0.0202	rbcL-accD	0.0177	trnA-1-trnA-2	0.0014
rps16-1-trnQ	0.0194	accD-psaI	0.0161	trnA-2-rrn23	0.0020
trnQ-psbK	0.0130	psaI-ycf4	0.0127	rrn23-rrn4.5	0.0011
psbK-psbI	0.0277	ycf4-cemA	0.0180	rrn4.5-rrn5	0.0017

Table S2. Continued

non-coding regions	Pi	non-coding regions	Pi	non-coding regions	Pi
psbI-trnS	0.0268	cemA-petA	0.0084	rrn5-trnR	0.0031
trnS-trnT-1	0.0202	petA-psbJ	0.0213	trnR-trnN	0.0051
trnT-1-trnT-2	0.0108	psbJ-psbL	0.0099	trnN-ycf1	0.0011
trnT-2-trnR	0.0204	psbE-petL	0.0177	ndhF-rpl32	0.0240
trnR-atpA	0.0226	petL-petG	0.0137	rpl32-trnL	0.0276
atpA-atpF-2	0.0113	petG-trnW	0.0188	ccsA-ndhD	0.0163
atpF-2-atpF-1	0.0110	trnW-trnP	0.0190	psaC-ndhE	0.0108
atpF-1-atpH	0.0124	trnP-psaJ	0.0179	ndhE-ndhG	0.0124
atpH-atpI	0.0134	psaJ-rpl33	0.0292	ndhG-ndhI	0.0175
atpI-rps2	0.0081	rpl33-rps18	0.0214	ndhI-ndhA-2	0.0102
rps2-rpoC2	0.0156	rps18-rpl20	0.0125	ndhA-2-ndhA-1	0.0173
rpoC2-rpoC1-2	0.0106	rpl20-rps12-1	0.0164	ndhH-rps15	0.0101
rpoC1-2-rpoC1-1	0.0121	rps12-1-clpP-3	0.0078	rps15-ycf1	0.0224
rpoB-trnC	0.0164	clpP-3-clpP-2	0.0098	ycf1-trnN	0.0161
trnC-petN	0.0174	clpP-2-clpP-1	0.0096	trnN-trnR	0.0052
petN-psbM	0.0117	clpP-1-psbB	0.0078	trnR-rrn5	0.0026
psbM-trnD	0.0257	psbB-psbT	0.0077	rrn5-rrn4.5	0.0017
trnD-trnY	0.0214	psbH-petB-1	0.0091	rrn4.5-rrn23	0.0011
trnE-trnT	0.0168	petB-1-petB-2	0.0097	rrn23-trnA-2	0.0020
trnT-psbD	0.0189	petB-2-petD-1	0.0101	trnA-2-trnA-1	0.0014
psbC-trnS	0.0199	petD-1-petD-2	0.0142	trnE-2-trnE-1	0.0036
trnS-psbZ	0.0143	petD-2-rpoA	0.0206	trnE-1-rrn16	0.0011
psbZ-trnG	0.0263	rps11-rpl36	0.0084	rrn16-trnV	0.0010
trnM-rps14	0.0072	rpl36-rps8	0.0262	trnV-rps12-2	0.0030
rps14-psaB	0.0099	rps8-rpl14	0.0092	rps12-2-rps12-3	0.0006
psaA-ycf3-2	0.0133	rpl16-2-rpl16-1	0.0196	rps7-ndhB-1	0.0024
ycf3-2-ycf3-1	0.0078	rpl16-1-rps3	0.0161	ndhB-1-ndhB-2	0.0010
ycf3-1-trnS	0.0056	rpl2-2-rpl2-1	0.0015	ndhB-2-trnL	0.0035
trnS-rps4	0.0078	rpl23-trnI	0.0027	trnL-ycf2	0.0037
rps4-trnT	0.0204	trnI-ycf2	0.0016	ycf2-trnI	0.0006
trnT-trnL-1	0.0104	ycf2-trnL	0.0037	trnI-rpl23	0.0027
trnL-1-trnL-2	0.0122	trnL-ndhB-2	0.0042	rpl2-1-rpl2-2	0.0015
trnL-2-trnF	0.0152	ndhB-2-ndhB-1	0.0010		
trnF-ndhJ	0.0205	ndhB-1-rps7	0.0024		

Table S3. Nucleotide diversity (Pi) values were calculated for 122 coding regions.

coding regions	Pi	coding regions	Pi	coding regions	Pi
psbA	0.0092	ycf4	0.0111	rps12-2	0.0000
trnK-2	0.0272	cemA	0.0075	trnV	0.0026
matK	0.0174	petA	0.0077	rrn16	0.0002
rps16-2	0.0210	psbJ	0.0178	trnE-1	0.0006
rps16-1	0.0198	psbF	0.0043	trnE-2	0.0011
trnQ	0.0181	psbE	0.0070	rrn23	0.0002
psbK	0.0187	petG	0.0126	rrn5	0.0031
atpA	0.0060	psaJ	0.0309	ycf1	0.0049
atpF-2	0.0112	rpl33	0.0246	ndhF	0.0113
atpF-1	0.0094	rps18	0.0160	trnL	0.0187
atpH	0.0127	rpl20	0.0152	ccsA	0.0186
atpI	0.0079	rps12-1	0.0132	ndhD	0.0110
rps2	0.0063	clpP-3	0.0098	psaC	0.0119
rpoC2	0.0098	clpP-2	0.0077	ndhE	0.0167
rpoC1-2	0.0072	clpP-1	0.0117	ndhG	0.0096
rpoC1-1	0.0056	psbB	0.0050	ndhI	0.0095
rpoB	0.0064	psbT	0.0066	ndhA-2	0.0088
petN	0.0139	psbN	0.0059	ndhA-1	0.0127
psbM	0.0112	psbH	0.0091	ndhH	0.0098
trnY	0.0219	petB-2	0.0050	rps15	0.0151
trnT	0.0249	petD-2	0.0145	ycf1	0.0261
psbD	0.0046	rpoA	0.0112	rrn5	0.0031
psbC	0.0041	rps11	0.0093	rrn23	0.0002
psbZ	0.0170	rpl36	0.0183	trnE-2	0.0011
trnG	0.0129	rps8	0.0158	trnE-1	0.0006
trnM	0.0038	rpl14	0.0064	rrn16	0.0003
rps14	0.0104	rpl16-2	0.0071	trnV	0.0026
psaB	0.0034	rps3	0.0141	rps12-2	0.0000
psaA	0.0031	rpl22	0.0202	rps7	0.0016
ycf3-1	0.0076	rps19	0.0119	ndhB-1	0.0014
rps4	0.0109	rpl2-2	0.0016	ndhB-2	0.0022
ndhJ	0.0066	rpl2-1	0.0030	trnL	0.0021
psbG	0.0063	rpl23	0.0038	ycf2	0.0025
ndhC	0.0134	ycf2	0.0025	rpl23	0.0038
atpE	0.0054	trnL	0.0032	rpl2-1	0.0030
atpB	0.0060	ndhB-2	0.0022	rpl2-2	0.0012
rbcL	0.0052	ndhB-1	0.0014		
accD	0.0103	rps7	0.0016		

Table S4. RSCU values of the chloroplast genome for *Megacarpaea megalocarpa* and other species of tribe Megacarpaeeae.

Amino acid	Codon	*Megacarpaea megalocarpa*	*Megacarpaea polyandra*	*Megacarpaea delavayi*	*Pugionium cornutum*	*Pugionium dolabratum*	*Pugionium pterocarpum*
Ter	UAA	1.77	1.80	1.73	1.73	1.73	1.76
	UAG	0.77	0.75	0.77	0.81	0.81	0.79
	UGA	0.46	0.45	0.50	0.46	0.46	0.45
Ala	GCA	1.12	1.12	1.14	1.11	1.10	1.12
	GCC	0.55	0.56	0.56	0.58	0.58	0.56
	GCG	0.41	0.41	0.40	0.43	0.43	0.43
	GCU	1.92	1.90	1.89	1.89	1.89	1.89
Cys	UGC	0.45	0.46	0.44	0.50	0.50	0.49
	UGU	1.55	1.54	1.56	1.50	1.50	1.51
Asp	GAC	0.38	0.37	0.38	0.37	0.37	0.39
	GAU	1.62	1.63	1.62	1.63	1.63	1.61
Glu	GAA	1.54	1.54	1.54	1.56	1.56	1.56
	GAG	0.46	0.46	0.46	0.44	0.44	0.44
Phe	UUC	0.61	0.61	0.61	0.60	0.60	0.60
	UUU	1.39	1.39	1.39	1.40	1.40	1.40
Gly	GGA	1.62	1.62	1.63	1.63	1.63	1.63
	GGC	0.41	0.40	0.40	0.40	0.40	0.40
	GGG	0.61	0.61	0.61	0.62	0.62	0.62
	GGU	1.36	1.37	1.36	1.35	1.35	1.35
His	CAC	0.48	0.47	0.48	0.48	0.48	0.47
	CAU	1.52	1.53	1.52	1.52	1.52	1.53
Ile	AUA	0.94	0.95	0.95	0.96	0.96	0.96
	AUC	0.52	0.51	0.52	0.51	0.51	0.51
	AUU	1.54	1.54	1.52	1.53	1.53	1.53
Lys	AAA	1.57	1.58	1.58	1.58	1.58	1.58
	AAG	0.43	0.42	0.42	0.42	0.42	0.42
Leu	CUA	0.81	0.82	0.81	0.82	0.82	0.81
	CUC	0.34	0.34	0.35	0.36	0.36	0.35
	CUG	0.33	0.33	0.33	0.35	0.35	0.36
	CUU	1.24	1.23	1.26	1.25	1.25	1.24
	UUA	2.16	2.17	2.14	2.12	2.12	2.13
	UUG	1.12	1.12	1.11	1.10	1.10	1.10
Met	AUG	1.00	1.00	1.00	1.00	1.00	1.00
Asn	AAC	0.46	0.46	0.46	0.47	0.46	0.47
	AAU	1.54	1.54	1.54	1.53	1.54	1.53

Table S4. Continued

Amino acid	Codon	*Megacarpaea megalocarpa*	*Megacarpaea polyandra*	*Megacarpaea delavayi*	*Pugionium cornutum*	*Pugionium dolabratum*	*Pugionium pterocarpum*
Pro	CCA	1.15	1.17	1.13	1.14	1.14	1.16
	CCC	0.72	0.70	0.74	0.71	0.71	0.72
	CCG	0.52	0.49	0.51	0.51	0.51	0.50
	CCU	1.61	1.63	1.63	1.64	1.64	1.63
Gln	CAA	1.58	1.58	1.57	1.58	1.58	1.58
	CAG	0.42	0.42	0.43	0.42	0.42	0.42
Arg	AGA	1.75	1.75	1.74	1.76	1.75	1.75
	AGG	0.58	0.59	0.60	0.58	0.59	0.58
	CGA	1.38	1.38	1.38	1.42	1.42	1.42
	CGC	0.42	0.42	0.45	0.42	0.42	0.43
	CGG	0.46	0.44	0.43	0.45	0.45	0.45
	CGU	1.41	1.41	1.41	1.38	1.38	1.38
Ser	AGC	0.37	0.36	0.35	0.35	0.35	0.35
	AGU	1.26	1.27	1.25	1.26	1.26	1.26
	UCA	1.19	1.21	1.22	1.21	1.21	1.22
	UCC	0.85	0.83	0.85	0.84	0.84	0.83
	UCG	0.55	0.55	0.56	0.55	0.55	0.55
	UCU	1.79	1.78	1.78	1.78	1.78	1.80
Thr	ACA	1.25	1.25	1.26	1.25	1.25	1.25
	ACC	0.69	0.68	0.69	0.72	0.72	0.71
	ACG	0.37	0.40	0.41	0.37	0.38	0.37
	ACU	1.68	1.68	1.64	1.67	1.66	1.67
Val	GUA	1.48	1.48	1.48	1.47	1.47	1.47
	GUC	0.48	0.47	0.50	0.49	0.49	0.48
	GUG	0.55	0.55	0.54	0.53	0.54	0.54
	GUU	1.49	1.50	1.48	1.51	1.50	1.51
Trp	UGG	1.00	1.00	1.00	1.00	1.00	1.00
Tyr	UAC	0.37	0.36	0.36	0.37	0.37	0.37
	UAU	1.64	1.64	1.64	1.63	1.63	1.63

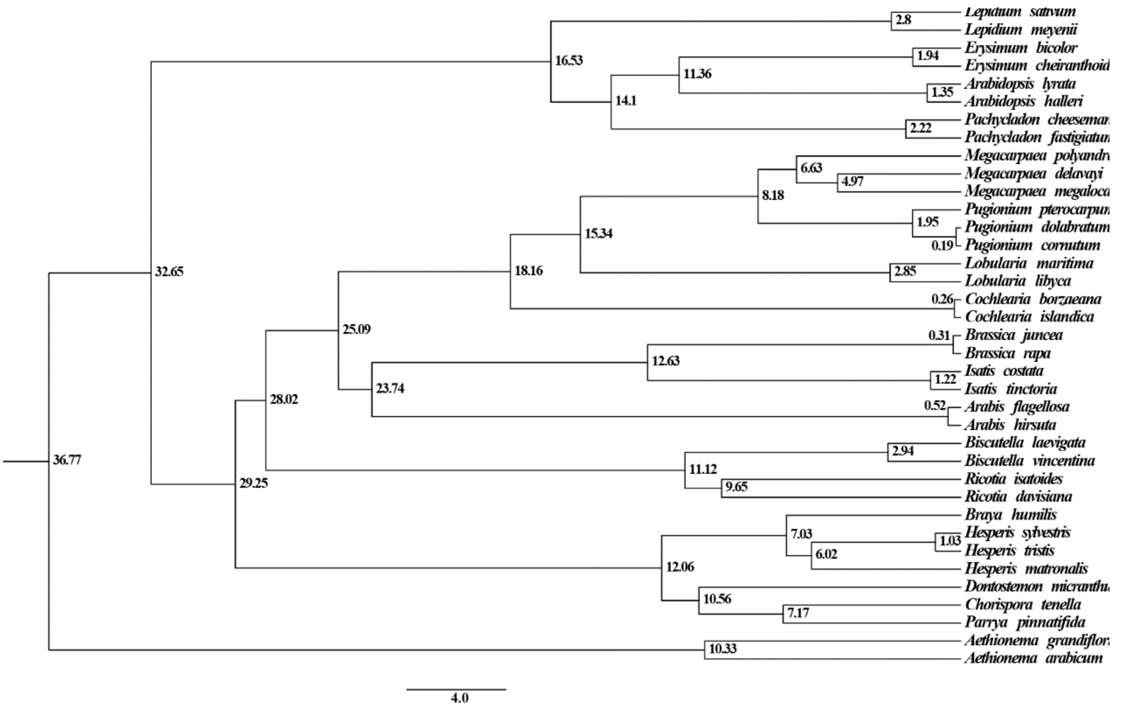

Figure S1. Divergence time (Million year ago) of Brassicaceae species inferred from the complete chloroplast genome data.

Disclaimer/Publisher's Note: The statements, opinions and data contained in all publications are solely those of the individual author(s) and contributor(s) and not of MDPI and/or the editor(s). MDPI and/or the editor(s) disclaim responsibility for any injury to people or property resulting from any ideas, methods, instructions or products referred to in the content.
